# Effect of local anaesthetic infiltration on postoperative pain after laparoscopic cholecystectomy: randomized clinical trial

**DOI:** 10.1093/bjsopen/zrac066

**Published:** 2022-05-27

**Authors:** Wikran Suragul, Apawee Tantawanit, Narongsak Rungsakulkij, Paramin Muangkaew, Pongsatorn Tangtawee, Somkit Mingphrudhi, Watoo Vassanasiri, Panuwat Lertsithichai, Suraida Aeesoa, Worapot Apinyachon

**Affiliations:** 1 Department of Surgery, Ramathibodi Hospital, Mahidol University, Bangkok, Thailand; 2 Department of Anesthesiology, Ramathibodi Hospital, Mahidol University, Bangkok, Thailand

## Abstract

**Background:**

Local anaesthetic infiltration is widely used to reduce pain after laparoscopic cholecystectomy (LC). This trial evaluated the effect of depth of local anaesthetic infiltration on postoperative pain reduction after LC.

**Methods:**

Patients undergoing elective LC between March 2018 and February 2019 were randomized into no infiltration, subcutaneous infiltration, and rectus sheath infiltration using bupivacaine. The primary outcome was 24-h postoperative cumulative morphine use, and the secondary outcomes were mean 24-h Numerical Rating Scale (NRS) for pain, and nausea, and vomiting. Subgroups were compared and multivariable analyses were performed.

**Results:**

Out of 170 eligible patients, 162 were selected and 150 patients were analysed: 48 in the no-infiltration group, 50 in the subcutaneous infiltration group, and 52 in the rectus sheath infiltration group. The groups had similar clinical features, although mean BMI was higher in the subcutaneous infiltration group (*P* = 0.001). The 24-h cumulative morphine use in the rectus sheath infiltration group was significantly lower than in the no-infiltration group (*P* = 0.043), but no difference was observed between the subcutaneous infiltration and no-infiltration groups (*P* = 0.999). One hour after surgery, the rectus sheath infiltration group had a significantly lower NRS score than the no-infiltration and subcutaneous infiltration groups respectively (*P* = 0.006 and *P* = 0.031); however, the score did not differ among the three groups at any of the time points from 2 h after the surgery. The incidence of nausea or vomiting was comparable among the three groups. Multivariable analysis documented that a lower dose of morphine use was associated with rectus sheath infiltration (*P* = 0.004) and diabetes (*P* = 0.001); whereas, increased morphine use was associate with age (*P* = 0.040) and a longer duration of surgery (*P* = 0.007).

**Conclusions:**

Local anaesthetic infiltration into the rectus sheath reduced postoperative cumulative morphine use and the immediate NRS score in patients undergoing LC; however, the pain scores were comparable 2 h after surgery.

**Registration number:**

TCTR20201103002 (http://www.thaiclinicaltrials.org).

## Teaser

Local anaesthetic infiltration into the rectus sheath reduced postoperative cumulative morphine use in patients undergoing laparoscopic cholecystectomy; however, infiltration into the subcutaneous tissue alone did not affect postoperative pain control.

## Introduction

Cholecystectomy is one of the most performed procedures globally. Laparoscopic cholecystectomy (LC) is now the standard because it is associated with less postoperative pain, faster recovery, and a shorter duration of hospital stay than an open approach^1^; however, postoperative pain is still an issue, especially during the first 24 h^2,3^. Many methods have been developed for postoperative pain control, including local anaesthetic infiltration, which is widely used and may hasten patient recovery; however, its effectiveness remains controversial. A meta-analysis published in 2014 showed that local anaesthetic wound infiltration reduces postoperative pain in the first 24 h, without increasing the incidence of serious adverse events, but the quality of the studies on which it was based was low and a great variety of anaesthetic infiltration methods were used^4^. Therefore, this randomized clinical trial (RCT) aimed to compare the effect of different depths of local anaesthetic infiltration on postoperative pain control in patients undergoing LC.

## Materials and methods

### Study design and sample size

This RCT was conducted at Ramathibodi Hospital, Mahidol University, between March 2018 and February 2019. The study was approved by the Committee of Research, Faculty of Medicine, Ramathibodi Hospital, Mahidol University (EC no. 10-61-04). Ramathibodi Hospital is a teaching hospital performing 200–250 cases of elective cholecystectomy per year.

### Participants

The study recruited patients who were scheduled for elective LC, aged 18–85 years, and ASA class I–IV. Patients who were diagnosed with liver cirrhosis, concurrent common bile duct cholelithiasis, acute pancreatitis, or critical illness; had a history of bupivacaine or morphine allergy; or needed surgical drain placement or conversion to open cholecystectomy were excluded from the study. The participants were informed about the procedure by a researcher and gave written consent on the admission date, before surgery. All patients were routinely admitted to the hospital 1 day before and discharged the day after the operation and were followed up at the outpatient clinic 1 week and 2 months after surgery.

Preoperative anaesthetic evaluation was performed and an intravenous (i.v.) patient-controlled analgesia (PCA) method was recommended by anaesthetists before surgery.

### Randomization and blinding

Participants were randomly allocated to three groups by way of computer-generated block randomization. Group 1 consisted of participants who did not receive local anaesthetic infiltration (no-infiltration group); group 2 consisted of participants who received local anaesthetic infiltration into the subcutaneous layer (subcutaneous infiltration group); and group 3 consisted of participants who received local anaesthetic infiltration into the rectus sheath and subcutaneous layer (rectus sheath infiltration group). In the subcutaneous infiltration group, 10 ml 0.5 per cent bupivacaine was injected at multiple sites into the subcutaneous layer of each surgical wound, and in the rectus sheath infiltration group, 5 ml 0.5 per cent bupivacaine was injected into the abdominal sheath of the infra-umbilical wound (∼10 mm in length) and 5 ml was injected to the subcutaneous layer of each wound, including the infra-umbilical wound.

Randomization numbers were written on pieces of paper and placed in opaque, sealed envelopes, which were opened in the operating room by nurses after each patient’s gall bladder and all the instruments were removed. The participants were blinded to their group allocation and the individuals who opened the envelopes did not participate in the postoperative pain control or observation. The surgeons were not blinded to the participants’ group allocations, but they did not participate in postoperative pain control or its assessment.

### Surgical technique

**Fig. 1:**
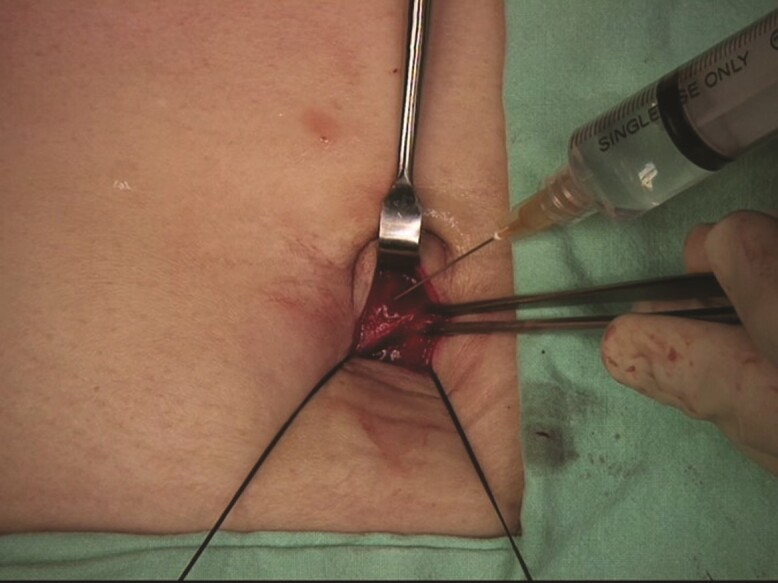
Administration of umbilical port infiltration

LC was performed by staff surgeons and residents, under close supervision. A total of three to five ports were used in each patient: a 10-mm trocar was inserted through the infra-umbilical incision by way of an open approach, then two to four more 5-mm trocars were inserted through incisions in the epigastric and right costal margin areas after creating a pneumoperitoneum. The intra-abdominal pressure was maintained between 12 and 15 mmHg during the procedure. Local anaesthetic infiltration was performed according to the group allocation at the end of surgery. For the rectus sheath infiltration group, the rectus sheath of the infra-umbilical incision was clearly identified by hanging sutures, which were placed at the time of trocar insertion. Then, 5 ml 0.5 per cent bupivacaine was directly infiltrated, resulting in swelling of the rectus sheath and the underneath peritoneum (*Fig. 1*).

### Anaesthesia and perioperative pain management

General anaesthesia was induced in all the participants. Fentanyl at 1.5 µg/kg was administered at the induction of anaesthesia and a minimal alveolar concentration of sevoflurane of 1–1.5 per cent was used to maintain anaesthesia; no further analgesia was provided. In the postoperative care unit (PACU), the pain score of the participants was evaluated with the Numerical Rating Scale (NRS) by the attending anaesthetists. Three milligrams of i.v. morphine were administered if the pain score of the participants was more than 3, and it was reassessed 15 min later. The i.v. PCA (IVAC^TM^ PCAM^TM^; CareFusion, Eysins, Switzerland) with morphine was commenced when the participant’s pain score was lower than 3. Postoperative PCA morphine administration consisted of a 1-mg bolus, a lockout interval of 5 min, and a 4-h limit of 40 mg. The number of times that the patients pressed the i.v. PCA button and when were recorded automatically by the device and this was reviewed by researchers.

### Outcomes

The primary outcome of the study was 24-h postoperative cumulative morphine use, and the secondary outcomes were mean pain intensity and postoperative nausea and vomiting. Pain intensity was assessed with the NRS by participants 0, 1, 2, 4, 8, 12, 16, 20, and 24 h after surgery. Participants were asked to rate their pain intensity from 0 to 10, with 0 representing no pain at all and 10 representing the worst pain imaginable. The immediate postoperative pain scores were assessed and recorded by the anaesthetists in the PACU and then ward nurses made their assessments. All individuals were blinded to the group allocation of the participants. The nausea and vomiting scores were assessed by the nurses in the PACU and wards.

All patient characteristics, operating factors, and operating outcomes were collected and factors associated with postoperative morphine use were analysed.

### Statistical analysis

The sample size was calculated to detect any significant difference in total morphine consumption among the three randomized groups at the 5 per cent level, with a power of 80 per cent. The mean total morphine consumption for the sample size calculation was obtained from a previous, similar research^5^, assumed to be applicable to the present study. In that study, the total morphine consumption at 48 h after LC for sham injection, injection of ropivacaine into the wound, and ropivacaine injection into the peritoneal cavity was 24, 17 and 21 mg respectively^5^. The common within-group variance (having the same value for all three groups), based on the s.d. obtained from the same study, was assumed to be 11 mg^2^. STATA version 14 (StataCorp, College Station, Texas, USA) was used to calculate the sample size, with the ‘power oneway’ command, obtaining 49 patients per group, a 10 per cent expected dropout in each group was added; on this basis, the final number of participants allocated per group was 54.

Statistical analysis was performed with STATA version 14 (StataCorp, College Station, Texas, USA). Continuous data are expressed as mean(s.d.) and categorical data are expressed as frequencies and percentages. Categorical data were analysed with a chi-squared test and continuous data were assessed with a one-way ANOVA or Kruskal–Wallis test. The factors associated with the level of morphine use were identified with a non-linear regression model that was based on an empirical mathematical relationship between morphine use and time. Estimates of these parameters and the associated 95 per cent confidence intervals (c.i.) were obtained with non-linear least squares, with estimated s.e. adjusted for clustering around individuals. *P* < 0.05 was considered to represent statistical significance and only significant predictors were included in the final model. The final equation of the multivariable analysis of the predictors associated with the total amount of morphine used was PCA_total_(*t*) = 6.651 + a1[(anaesthetic group)(optime)(diabetes millitus)] x (1-exp^(−0.375)*t* +(–0.003)age^).

## Results

**Fig. 2:**
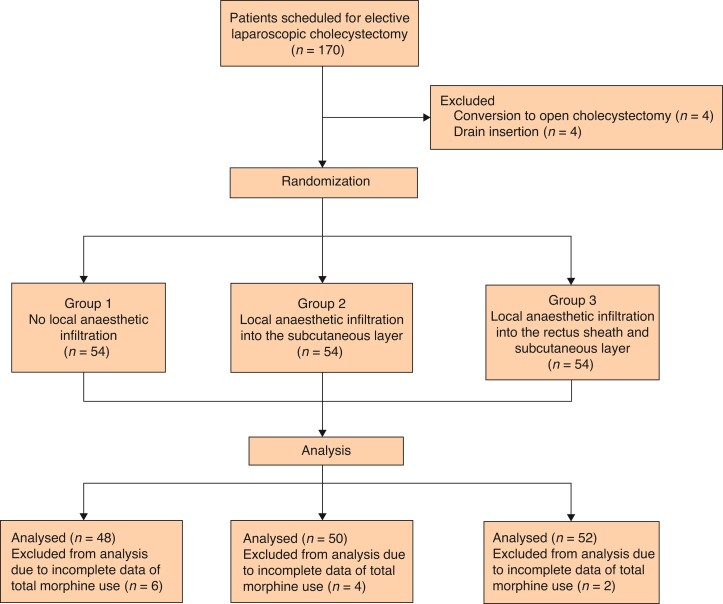
Flow chart of the study design

A total of 170 patients underwent LC during the study interval, of whom 4 patients who required conversion to open cholecystectomy and 4 patients who required surgical drain insertion were excluded, such that 162 participants were randomized to the three groups. Overall, 12 participants dropped out of the study due to incomplete data on postoperative morphine use. The total number of participants analysed in the study was 150 (*Fig. 2*). The characteristics of the participants, operating factors, and operating outcomes are summarized in *Tables 1* and *2*. The characteristics and operating factors of the three groups were similar: there were no differences in sex distribution, age, ASA physical status, distribution of diagnoses, number of surgical incisions, pneumoperitoneum pressure, duration of surgery, or estimated blood loss; however, mean BMI was higher in the subcutaneous infiltration group (*P* = 0.001). There were also no differences in the incidence of perioperative complications. The number of participants who required analgesia in the PACU was highest in the no-infiltration group and lowest in the subcutaneous infiltration group, but the differences between the groups were not statistically significant (*P* = 0.198). The duration of hospital stay was not different among the three groups due to the routine admission protocol of our hospital; consequently, this variable was not analysed as the outcome.

**Table 1:** Characteristics of the participants and operating factors

Parameter	All participants (*n* = 150)	Group 1 No local infiltration (*n* = 48)	Group 2 Subcutaneous infiltration (*n* = 50)	Group 3 Rectus sheath and subcutaneous infiltration (*n* = 52)	*P*
**Sex**					
Male	42 (28.00)	9 (18.75)	17 (34.00)	16 (30.77)	0.209
Female	108 (72.00)	39 (81.25)	33 (66.00)	36 (69.23)	
**Age (years), mean(s.d.)**	55.87 (14.28)	52.75 (14.23)	55.80 (13.80)	58.83 (14.42)	0.951
**BMI, mean(s.d.)**	25.43 (5.40)	25.09 (6.46)	26.01 (5.73)	25.19 (3.83)	0.001
**ASA physical status class**					
I	33 (22.00)	12 (25.00)	12 (24.00)	9 (17.31)	0.882
II	76 (50.67)	22 (45.83)	25 (50.00)	29 (55.77)	
III	39 (26.00)	14 (29.17)	12 (24.00)	13 (25.00)	
IV	2 (1.33)	0	1 (2.00)	1 (1.92)	
**Co-morbidity**					
Diabetes mellitus	29 (19.33)	9 (18.75)	11 (22.00)	9 (17.31)	0.829
Hypertension	68 (45.33)	23 (47.92)	20 (40.00)	25 (48.08)	0.650
Dyslipidaemia	55 (36.67)	17 (35.42)	17 (34.00)	21 (40.38)	0.781
Obesity	16 (10.67)	5 (10.42)	6 (12.00)	5 (9.62)	0.925
Thalassemia	9 (6.00)	4 (8.33)	4 (8.00)	1 (1.92)	0.299
**Diagnosis**					
Symptomatic gallstone	106 (70.67)	34 (70.83)	39 (78.00)	33 (63.46)	0.288
Gallbladder polyp	14 (9.33)	3 (6.25)	6 (12.00)	5 (9.62)	
Chronic cholecystitis	25 (16.67)	8 (16.67)	5 (10.00)	12 (23.08)	
Gallstone pancreatitis	5 (3.33)	3 (6.25)	0	2 (3.85)	
**Number of incisions**					
3	22 (14.67)	5 (10.42)	10 (20.00)	7 (13.46)	0.373
4	127 (84.67)	43 (89.58)	39 (78.00)	45 (86.54)	
5	1 (0.67)	0	1 (2.00)	0	
**Intra-abdominal pressure (mmHg), median (i.q.r.)**	12 (12, 14)	12 (12, 14)	12 (12, 15)	12 (12, 15)	0.854*
**Duration of surgery (min), mean(s.d.)**	75.13 (25.52)	74.37 (25.15)	74.90 (26.48)	76.06 (25.38)	0.929
**EBL (ml), median (i.q.r.)**	10 (5, 20)	10 (5, 20)	5 (5, 10)	10 (5, 20)	0.166*
**Intraoperative use of fentanyl**	150 (100)	48 (100)	50 (100)	52 (100)	—
**Intraoperative anti-emetic**					
No	7 (4.67)	1 (2.08)	2 (4.00)	4 (7.69)	0.505
Yes	143 (95.33)	47 (97.92)	48 (96.00)	48 (92.31)	

i.q.r., interquartile range; EBL, estimated blood loss. *Kruskal–Wallis test. Values are *n* (%) unless otherwise indicated.

**Table 2:** Operating outcomes

Parameter	All participants (*n* = 150)	Group 1 No local infiltration (*n* = 48)	Group 2 Subcutaneous infiltration (*n* = 50)	Group 3 Rectus sheath and subcutaneous infiltration (*n* = 52)	*P*
**Perioperative complications**					
No	145 (96.67)	47 (97.92)	49 (98.00)	49 (94.23)	0.620
Yes	5 (3.33)	1 (2.08)	1 (2.00)	3 (5.77)	
**Clavien–Dindo classification**					
I	3 (2.00)	0	1 (2.00)	2 (3.84)	
II	2 (1.33)	1 (2.1)	0	1 (1.92)	
III	0	0	0	0	
IV	0	0	0	0	
V	0	0	0	0	
**PACU analgesia**					
No	81 (54.00)	22 (45.83)	26 (52.00)	33 (63.45)	0.198
Yes	69 (46.00)	26 (54.17)	24 (48.00)	19 (36.54)	
**PACU anti-emetic**					
No	124 (82.67)	39 (81.25)	44 (88.00)	41 (78.85)	0.452
Yes	26 (17.33)	9 (18.75)	6 (12.00)	11 (21.15)	
Ondansetron	7 (4.67)	1 (2.08)	2 (4.00)	4 (7.69)	
Metoclopramide	20 (13.33)	8 (16.67)	5 (10.00)	7 (13.46)	
Dimenhydrinate	1 (0.67)	0	0	1 (1.92)	
**PACU NV score**					
None	124 (82.67)	39 (81.25)	44 (88.00)	41 (78.85)	0.410
Mild	24 (16.00)	8 (16.67)	5 (10.00)	11 (21.15)	
Severe	2 (1.33)	1 (2.08)	1 (2.00)	0	
**Ward NV**					
No	132 (88.59)	40 (85.11)	44 (88.00)	48 (92.31)	0.524
Yes	17 (11.41)	7 (14.89)	6 (12.00)	4 (7.69)	

PACU, postoperative care unit; NV, nausea and vomiting. Values are *n* (%) unless otherwise indicated.

### Postoperative analgesic drug use

**Fig. 3:**
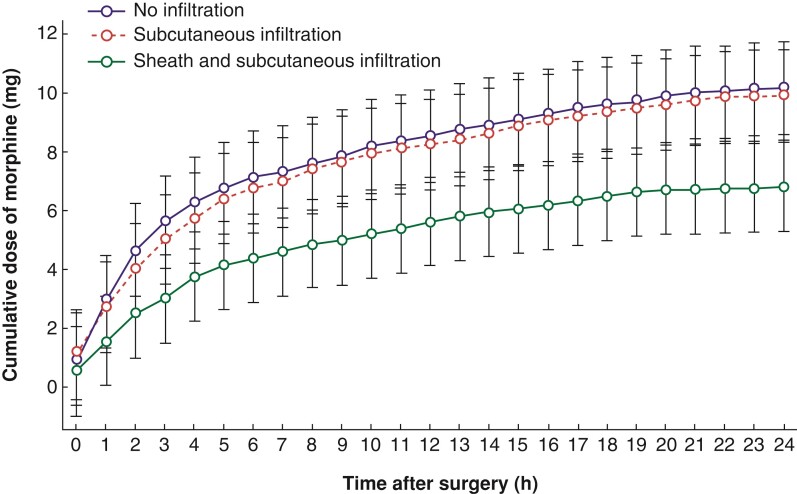
Cumulative dose of morphine administered via intravenous patient-controlled analgesia

The amount of morphine administered after surgery was highest in the no-infiltration group and lowest in the rectus sheath infiltration group at every time point. The mean 24-h cumulative morphine use by participants in the rectus sheath infiltration group was 3.36 mg lower than that by participants in the no-infiltration group (*P* = 0.043) and tended to be lower than that by participants in the subcutaneous infiltration group (by 3.11 mg, *P* = 0.065); however, no significant difference was found in the 24-h postoperative morphine use by the no-infiltration and the subcutaneous infiltration groups (*P* = 0.999) (*Fig. 3*).

### Postoperative pain

**Fig. 4:**
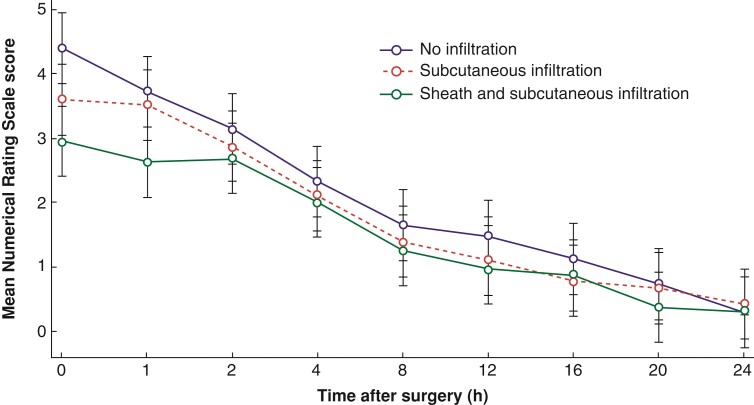
Postoperative pain score

The pain intensity was highest immediately after surgery, then gradually decreased. The rectus sheath infiltration group had significantly lower NRS score than the no-infiltration group (*P* = 0.014) immediately following surgery. One hour after surgery, the rectus sheath infiltration group had significantly lower NRS scores than the no-infiltration group (*P* = 0.006) and the subcutaneous infiltration group (*P* = 0.031); however, no difference in NRS scores was observed between the no-infiltration group and the subcutaneous infiltration group at either time point. The NRS score did not differ among the three groups at any of the time points from 2 h after surgery (*Fig. 4*).

### Postoperative nausea and vomiting score

Most of the participants did not have nausea and vomiting after the operation and no differences in the incidence of postoperative nausea and vomiting was observed among the three groups. (*Table 2*)

### Factors related to postoperative morphine consumption

*Table 3* shows the results of univariable and multivariable analyses of variables associated with postoperative morphine consumption after LC. In the multivariable analyses, the rectus sheath infiltration group was strongly associated with a significantly lower dose of morphine use than the other two groups and diabetes was also associated with a lower postoperative morphine requirement. Furthermore, age negatively correlated with postoperative morphine use and a longer duration of surgery was strongly positively associated with high postoperative morphine use.

**Table 3:** Factors associated with the total amount of morphine used

Parameter	Univariable analysis		Multivariable analysis	
	Coefficient (95% c.i.)	*P*	Coefficient (95% c.i.)	*P*
**Group**				
No-infiltration	1		1	
Subcutaneous infiltration	−0.317 (−3.06 to 2.42)	0.819	−0.165 (−2.79 to 2.46)	0.901
Sheath and subcutaneous infiltration	−3.385 (−5.77 to −1.00)	0.006	−3.451 (−5.78 to −1.12)	0.004
**Sex**				
Male	1			
Female	−0.073 (−0.26 to 0.11)	0.438		
**Age (years)**	−0.004 (−0.01 to −0.00)	0.019	−0.003 (−0.01 to −0.00)	0.040
**BMI (kg/m^2^)**	−0.003 (−0.01 to 0.00)	0.551		
**ASA physical status class**				
I	1			
II	−0.144 (−0.33 to 0.04)	0.134		
III	−0.244 (−0.38 to −0.10)	0.001		
IV	−0.255 (−0.40 to −0.11)	0.001		
**Co-morbidity**				
Diabetes mellitus	−0.156 (−0.21 to −0.11)	<0.001	−3.541 (−5.69 to −1.39)	0.001
Hypertension	−0.185 (−0.24 to −0.13)	<0.001		
Dyslipidaemia	−0.034 (−0.18 to 0.12)	0.653		
Obesity	0.121 (−0.17 to 0.41)	0.420		
Thalassemia	0.506 (−0.11 to 1.12)	0.108		
**Diagnosis**				
Symptomatic gallstone	1			
Gallbladder polyp	−0.116 (−0.19 to −0.05)	0.002		
Chronic cholecystitis	0.124 (−0.12 to 0.36)	0.313		
Gallstone pancreatitis	−0.144 (−0.20 to −0.08)	<0.001		
**Number of incisions**				
3	1			
4	0.076 (−0.06 to 0.21)	0.276		
5	0.280 (0.01 to 0.55)	0.045		
**Duration of surgery (min)**	0.047 (0.01–0.08)	0.015	0.052 (0.01 to 0.09)	0.007
**EBL (ml)**	0.007 (0.00 to 0.01)	0.016		

EBL, estimated blood loss.

## Discussion

Numerous studies have investigated the effectiveness of local anaesthetic infiltration for postoperative pain control, but few have assessed the effect of the depth of anaesthetic infiltration. The present study documented that local anaesthetic infiltration into the rectus sheath reduces postoperative pain, assessed with the cumulative morphine consumption and NRS score. The total amount of morphine administered by the rectus sheath infiltration group was significantly lower than that administered by the no-infiltration group and tended to be lower than that administered by the subcutaneous infiltration group. Additionally, the pain score was lower in the rectus sheath infiltration group than in the other two groups during the early postoperative interval, whereas anaesthetic infiltration into the subcutaneous tissue alone did not have an effect on postoperative pain control.

The first RCT investigating the effect of depth of anaesthetic infiltration around ports for LC was conducted in 1996, and showed that the injection of local anaesthetic around such ports at the level of the parietal peritoneum reduced postoperative pain *versus* standard subcutaneous tissue injection^6^. Specifically, the study showed significantly lower pain scores 6 and 18 h after the procedure, but the total dose of analgesic required did not differ between the groups. The study concluded that the injection of bupivacaine at the level of the parietal peritoneum reduced immediate postoperative pain.

The depth of injection differed between the present study and that of the aforementioned study, but the relevant layers are very close and there is likely to be some overlap between the areas of infiltration. Indeed, a swelling of both the abdominal sheath and the parietal peritoneum during the infiltration in most of the participants in the present study was observed.

In the present study, a significant difference in pain score was found immediately and 1 h after surgery, which is not consistent with the findings of the first trial, perhaps because of the different methods of postoperative pain control used. An intramuscular injection of pethidine and oral naproxen was used for postoperative pain control in the previous study, whereas we used i.v. PCA with morphine, which is a widely used, and effective method of postoperative pain control^7^. This method seemed to provide effective pain control because there was a low mean pain score (lower than 3) in all the participants from 3 h after surgery; however, the duration of the pain reduction induced by local anaesthesia differed among the many previous studies. Some studies demonstrated an analgesic effect only during the early postoperative interval (0–6 h)^3,8–10^, whereas others showed a longer-lasting effect (12–24 h)^11,12^. Various techniques, sites of infiltration, doses of anaesthetic, and postoperative analgesia regimens were used in these studies, which would have affected the duration of its effect.

Previous studies have also yielded inconsistent findings regarding the effects of anaesthetic infiltration around LC incisions, but various depths of portal wound infiltration were used in these studies. The studies in which anaesthetic was infiltrated into all the layers of the abdominal wall, including the subcutaneous tissue, fascia, and parietal peritoneum, showed significant reductions in postoperative pain^3,8,10,12^, whereas the study in which bupivacaine was infiltrated around trocar sites into the subcutaneous tissue alone showed no significant effect on postoperative pain reduction *versus* placebo^13^. These findings are consistent with the present finding that subcutaneous infiltration did not significantly affect postoperative pain control, whereas infiltration into the rectus sheath had a beneficial effect.

In the present study, bupivacaine was infiltrated into the rectus sheath and into the subcutaneous layer at the sub-umbilical incision, whereas at the other three incisions, bupivacaine was infiltrated into the subcutaneous tissue alone. This approach was chosen because the sub-umbilical incision is large and likely causes more pain than the other incisions; we routinely used an open technique for the sub-umbilical trocar insertion, where the layers of the abdominal wall can be clearly identified, such that accurate anaesthetic infiltration can be easily performed; and this technique is simple, requiring no additional resources, and could therefore be easily used in clinical practice.

The timing of anaesthetic infiltration has been investigated in previous studies; however, the result are inconsistent. Several studies demonstrated that preoperative incisional infiltration had better effect on postoperative pain control than postoperative infiltration^9,14^ but several studies demonstrated a contradictory result^6,15^. Therefore, the best timing of infiltration is still debatable; however, an advantage of postoperative infiltration would be the precision of the injection of anaesthesia into the particular layers of the abdominal wall, especially at the umbilical wound, because the skin is already open.

Other methods of postoperative pain control have been proposed. Transversus abdominis plane (TAP) block showed beneficial effects on postoperative pain control in LC compared with placebo^16,17^. Several RCTs have been conducted to compare the effects of TAP block with local anaesthetic infiltration; however, they showed contradictory results^18–20^. The previous RCT evaluated additional effects of TAP block in participants who received standard port site infiltration; the study concluded that TAP block did not give additional pain relief or other favourable outcomes^21^. Heterogeneous techniques of the TAP block and local anaesthetic infiltration were observed among the published studies. To compare the TAP block with local anaesthetic infiltration, more studies and standardized techniques are required. Continuous wound infusion was another method that had effects on postoperative pain reduction in various types of surgery^22^. The previous RCT compared perioperative continuous ropivacaine wound infusion with normal saline wound infusion and showed that the ropivacaine group had better pain control than the normal saline group up to 4 h after surgery^23^; however, there have been no studies comparing continuous wound infiltration with local anaesthetic infiltration to date.

The meta-analysis demonstrated that a low-pressure pneumoperitoneum was safe and resulted in reduced postoperative pain, rate of analgesic used, and duration of hospital stay^24^. Low-pressure cholecystectomy was safely performed by an experienced surgeon^25^. In our centre, low-pressure cholecystectomy was selectively performed but the data were not included in the present study. A combination of low-pressure pneumoperitoneum and local anaesthetic infiltration may provide a greater effect on postoperative pain reduction and may be an area for future studies.

According to the present findings, older patients require a lower dose of morphine for postoperative pain control than younger patients, which is consistent with the results of another recent study of LC, showing that the postoperative pain rating decreased with increasing age^26^. A systematic review published in 2009 showed that age negatively correlates with postoperative analgesic use and pain intensity. Many factors may contribute to this finding, including blunted peripheral nociceptive function, postoperative confusion and under-reporting of pain, high sensitivity to analgesics, and pharmacokinetic change, such as changes in drug metabolism or elimination^27^.

A prolonged duration of surgery was associated with a higher total dose of morphine. A long duration is a common feature of complex surgery and challenging cases, and is associated with more manipulation in the surgical field and greater tissue trauma around the trocar sites. Additionally, carbon dioxide, which is used for creating the pneumoperitoneum, promotes postoperative pain as a consequence of the build-up and accumulation of carbonic and lactic acid at the peritoneal membrane^28^; therefore, prolonged carbon dioxide insufflation may cause more postoperative pain and result in the use of more analgesics. The experience of surgeons influences the duration of surgery, which might also affect postoperative pain and the analgesic used; however, no study has addressed this issue to date and future studies are required.

Diabetes mellitus (DM) was associated with a lower requirement for postoperative morphine in the present study: participants with diabetes used 3.54 mg less morphine in total than those without. A previous study of patients undergoing cardiac surgery showed that those with DM had lower postoperative pain scores than those without, which the authors ascribed to poorer sensation, secondary to peripheral neuropathy^29^.

The present study had several limitations. First, visceral pain was not controlled with a specific procedure, and therefore this type of pain may have contributed to the overall postoperative pain score and requirement for morphine. Second, analgesia was provided in the form of a combination of intraoperative fentanyl and postoperative PCA morphine, and therefore did not include multimodal analgesia, such as i.v. dexamethasone, paracetamol, non-steroid anti-inflammatory drugs (NSAIDs), or nefopam. The assumption made was that the pain score and morphine consumption would be lower if these analgesics had also been used. In the pilot study, most of the patients had low postoperative pain scores, even in some patients who did not receive perioperative NSAIDs. Additionally, several previous studies showed that perioperative NSAIDs provided no significant effect on postoperative pain relief and opioid consumption after LC^30,31^. To accurately investigate effects of local anaesthetic infiltration on postoperative morphine use without being obscured by the effects of other types of analgesia, other analgesic drugs were omitted, including NSAIDs. Fourth, the patients were routinely admitted 1 day before and discharged 1 day after the operation, so the duration of hospital stay, which is a key outcome, could not be evaluated in the present study. Last, we used a total of 10 ml 0.5 per cent bupivacaine to infiltrate the surgical wounds or rectus sheath, but in a future study, the effects of a larger amount of local anaesthetic should also be investigated.

Based on these findings, deep local anaesthetic infiltration into the rectus sheath lowers immediate (within 2 h) postoperative pain scores and use of morphine during the first postoperative day after LC but subcutaneous infiltration alone has no effect on pain control.

## Supplementary Material

zrac066_Supplementary_data

## Data Availability

The data that support the findings of this study are available from the corresponding author upon reasonable request.
